# Do Repeated Sprints Affect the Biceps Femoris Long Head Architecture in Football Players with and without an Injury History?—A Retrospective Study

**DOI:** 10.3390/biology12010096

**Published:** 2023-01-08

**Authors:** Ricardo Pimenta, Hugo Antunes, Tomás Lopes, António Veloso

**Affiliations:** 1 CIPER, Faculdade de Motricidade Humana, Universidade de Lisboa, 1649-004 Cruz Quebrada-Dafundo, Portugal; 2 Department of Biochemistry, King’s College London, London WC2R 2LS, UK

**Keywords:** biceps femoris long head, architecture, hamstring strain injury, repeated sprint ability, fatigue

## Abstract

**Simple Summary:**

Football is a sport which imposes an intermittent type of physical exertion, and one of the main physical demands of the game is sprinting, covering mostly short distances on repeated occasions. Sprinting imposes maximum demands, especially on the hamstrings muscle group; this activity frequently contributes to the occurrence of injury to the biceps femoris long head muscle, particularly in the later stages of the game, suggesting that fatigue may play an important role. After the injury, sprint performance and strength might be reduced, and muscle morphology can be altered even after the player returns to competition. Therefore, the aim of the study was to verify the effect of a fatiguing task on sprint performance, the biceps femoris long head muscle morphology and the hamstring muscle force capacity. Moreover, a comparison was performed between injured players and healthy controls and injured versus contralateral limbs. Muscle morphology was assessed through ultrasound techniques, while muscle force was estimated using a dynamometry assessment. It was concluded that injured players tend to be faster than the healthy control group and display shorter biceps femoris long head fascicle length when compared with the contralateral limb and healthy controls.

**Abstract:**

The aim of this study was to compare the biceps femoris long head (BFlh) architecture between football players with (twelve) and without (twenty) history of BFlh injury before and after a repeated sprint task. Fascicle length (FL), pennation angle (PA) and muscle thickness (MT) were assessed at rest and in the active condition before and after the repeated sprint protocol. Athletes with previous BFlh injury showed shorter FL at rest (*p* = 0.014; η^2^_p_ = 0.196) and active state (*p* < 0.001; η^2^_p_ = 0.413), and greater PA at rest (*p* = 0.002; η^2^_p_ = 0.307) and active state (*p* < 0.001; η^2^_p_ = 0.368) before and after the task. Intra-individual comparisons showed that injured limbs have shorter FL at rest (*p* = 0.012; η^2^_p_ = 0.519) and in the active state (*p* = 0.039; η^2^_p_ = 0.332), and greater PA in passive (*p* < 0.001; η^2^_p_ = 0.732) and active conditions (*p* = 0.018; η^2^_p_ = 0.412), when compared with contralateral limbs. Injured players, at rest and in the active condition, display shorter BFlh FL and greater PA than contralateral and healthy controls after repeated sprints. Moreover, the BFlh of injured players presented a different architectural response to the protocol compared with the healthy controls.

## 1. Introduction

Hamstring strain injury (HSI) is the most common non-contact muscle injury in high-speed running sports [1,2,3,4,5,6,7], with a particularly high injury rate in football. It accounts for 37% of all muscle injuries [8], with a recurrence of 12–33% [7,9,10], which implies a significant financial cost for clubs. Indeed, the average cost of having a professional first team player injured for 1 month is approximately EUR 500.000 in European football leagues [11]. Due to these implications, HSI has drawn the attention of sports scientists and researchers. However, until today, the most consistent risk factor is recent (same season) HSI and previous HSI, although other factors such as old age, anterior cruciate ligament injury history and calf injury history have been suggested more recently [12]. It should also be noted that injuries occurred in the later stages of each half [8], suggesting that fatigue could also be a potential risk factor.

One way to induce fatigue is through repeated sprints [13], with the sprint having been identified as the most common mechanism for sustaining an HSI [4], particularly in the biceps femoris long head (BFlh) [14]. Despite limited evidence, the FL of the BFlh has been associated with a risk of HSI [12], since previous studies have reported differences in BFlh architecture between previously injured and uninjured players [15,16,17]. The potential physiological hypothesis is that the BFlh and the semimembranosus (SM) are two muscles with shorter fibers and greater PA, indicating a lower excursion capacity [18]. In terms of injury, muscles with greater fiber lengths have a greater lengthening capacity than muscles consisting of shorter fibers [19]. Therefore, when all hamstrings contract as they lengthen (eccentrically), muscles with shorter fibers will undergo higher strain, potentially predisposing the BFlh and SM to greater injury risk [20]. Curiously, these two muscles are the most affected in HSI, with most injuries in the BFlh occurring during sprint in the swing late phase [14,21] and in the SM during stretching-type movements (i.e., during movements with large joint excursions) [22,23]. Therefore, the rationale exists to support the hypothesis that shorter fascicles are a risk factor for HSI. Indeed, it has been reported that having shorter BFlh fascicles increased the risk of a future HSI, and low levels of eccentric knee flexor strength increased the risk of an HSI occurring in the subsequent season [15]. Moreover, Timmins et al. (2016) reported that the increased risk associated with increasing age and a history of HSI can be mitigated by higher levels of eccentric knee flexor strength and longer BFlh fascicles. It should be noted that muscle architecture is altered significantly when the muscle is active [19,24], with the serial sarcomeres number (which determines the FL) playing a role in contraction velocity and shortening capacity, while sarcomeres in parallel (linked to the muscle cross-sectional area and positively correlated with PA) dictate the force output potential [25]. Therefore, players with previous muscle damage, specifically HSI, would be expected to display a shorter FL and greater PA than healthy players with no previous HSI and compared with the contralateral (uninjured) limb due to a reduced sarcomere number in series. However, it should be interesting to analyze the muscle architecture after an ecological task with the most common injury mechanism, the sprint, as task specificity could induce different outcomes [26]. 

The purpose of this study was to compare the acute effects of a repeated sprint protocol on resting and active state BFlh architecture between professional football players with and without BFlh injury. Architecture was determined using ultrasound B-mode. We hypothesized that injured players would have shorter fascicles in comparison with their contralateral limbs and the uninjured players. Secondly, we hypothesized that the sprint task will induce changes on BFlh architecture with shorter FL after the task in the injured players compared with the healthy controls and contralateral limbs.

## 2. Materials and Methods

Thirty-two male professional football players (age = 25.03 ± 3.72 years, height = 1.79 ± 0.07 m, body mass = 74.69 ± 8.67 kg) participated in the present study divided in two groups. The healthy control group was composed of twenty professional football players without BFlh strain injury history. The injury group was composed of twelve professional football players with a unilateral BFlh strain injury history within the last 11.7 ± 7.4 months (ranging between 2 and 23 months). Lower limb injury history was registered by a physiotherapist, obtained through interview and by using musculoskeletal injury information recorded in the club’s medical department. Each injury diagnosis was based on MRI, ultrasound or palpation examination. All the players returned to pre-injury levels of training and competition and were recruited to assess the differences in architecture between their previously injured and uninjured BFlh. All participants read and signed an informed consent form before participation in the study. The Ethical Committee at the Faculty of Human Kinetics at the University of Lisbon approved the study (#5/2021).

### 2.1. Dynamometry

The knee flexor linear torque was measured at a sampling rate of 1000 Hz using a custom-made equipment. Participants were placed in a prone position, with the hips in neutral anatomical position, knees flexed at 30° (0° = full extension) and the ankle in 15° of plantar flexion, as hamstrings are in minimal passive tension [27]. The feet were fixed in a foot holder which contained a force transducer (Model STC, Vishay Precision, Malvern, PA, USA) at the heel level to collect the linear force perpendicular to the leg orientation and with the ankle at 90°. Force data was amplified (Model UA73.202, Sensor Techniques, Cowbridge, UK), digitally converted (USB-230 Series, Measurement Computing Corporation Norton, MA, USA), recorded using the DAQami software (v4.1, Measurement Computing Corporation, Norton, MA, USA) and multiplied by the perpendicular distance between the force transducer center and the femoral lateral condyle in order to estimate the knee torque. Visual feedback of force production was provided to individuals during the assessments.

### 2.2. BFlh Architecture Assessment

Two identical ultrasound scanners (Aixplorer, v11; Supersonic Imagine, Aix-en-Provence, France) were used to assess the FL, muscle thickness (MT), and PA of the BFlh which were determined from ultrasound images taken along the longitudinal axis of the muscle belly using an ultrasound transducer (SL10-2, 38 mm, 2–10 MHz. Super Linear, Vermon, Tours, France). 

The scanning site was determined with the considerations previously reported [28], at approximately 50% of the femur length (distance between the lateral femoral condyle and greater trochanter). To ensure a stable measure of the muscle architecture during the contractions and the measurement in the same region, a plastic cast was fixed to the skin using bi-adhesive tape due to the importance of keeping the same region of interest (ROI) over time for ultrasound imaging, as errors in probe placement could strongly influence the muscle architecture outcomes.

To minimize the time between the sprint task and the measurements of both limbs, the images were acquired by two examiners to enable the assessment of both limbs simultaneously. The most experienced examiner, having demonstrated their reliability in a previous study analyzing BFlh architecture [28], determined the region of interest by placing the casts, minimising the influence of the other examiner.

### 2.3. Protocol

Participants visited the track and field high performance center of the Jamor indoor facility. Both limbs were tested for neuromuscular and muscle architecture parameters with the participants in the previously described position. Testing began with participants resting for ~30 min to allow for body fluid shift stabilization [29,30]. Then, two videos were recorded for each BFlh with the individuals at rest. A standardized warm-up for force production was performed (i.e., 20 submaximal and 3 maximal knee flexions), followed by two 3-s MVIC trials with 30 s recovery between trials. Based on the MVIC highest peak torque (PT), individuals familiarized themselves with the 20% of MVIC trials using visual feedback. Each trial lasted ~40 s. The videos during active state were taken twice at 20% of MVIC for each BFlh. After active measures, a standardized warm-up protocol was conducted. Immediately after the warm-up, a 10 × of 30 m repeated sprint task with 30 s of rest between sets was performed, with participants positioned 1 m behind the photocells. The same active and rest measurements (in this order) were conducted after the repeated sprint task, followed by two MVIC at the end of the protocol.

### 2.4. Data Processing

**Figure 1:**
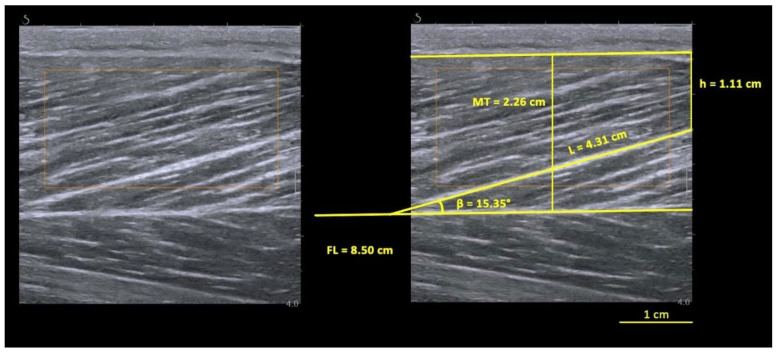
Biceps femoris long head (BFlh) architecture, measured at pre-task active condition. L, observable fascicle length; h, height; β, pennation angle; MT, muscle thickness; FL, fascicle length.

Prior selection of the sonograms matching the criteria explained below was performed for each video recorded. All sonograms were digitized using ImageJ software (NIH, 1.47v, Bethesda, MD, USA), and FL, PA and MT were determined from each sonogram. The researcher was blinded to the outcome during the digitization process. As only part of fascicle could be visualized during static-image assessments, the linear extrapolation method was used to estimate the non-visible portion of the fascicle (15) using the following Equation (1):

FL = L + (h/sinβ)(1)

where L is the observable fascicle length from the mid-muscle aponeurosis to the most visible endpoint, h is the perpendicular distance between the superficial aponeurosis and the fascicle’s visible distal end-point and β is the angle between the fascicle (drawn linearly to the most distal point) and the superficial aponeurosis, as in Figure 1. The MT was measured as the distance between the superficial and the mid-muscle aponeurosis measured at the ROI site. For each condition, two videos were recorded, and a frame was selected from each video. This was conducted in pre- and post-task instants (totaling 8 images for each limb). Three fascicles were selected in each image, meeting the criterion that a reasonable portion of the fascicle (~25% or more of the total estimated length) must have been visible within the ultrasound transducer’s field of view to be included in the present study [31].

### 2.5. Statistical Analysis

Data are presented as mean ± standard deviation. Data analysis was performed using IBM SPSS Statistics 27.0 (IBM Corporation, Armonk, NY, USA). Normality of data distribution was confirmed using the Shapiro–Wilk test. A paired sample t-test was performed for the descriptive statistics: age, height, and weight were calculated and comparisons between players with HSI history and healthy controls were made. An inter-individual comparison was performed between injured players and healthy controls. With respect to the effects of the sprint task, their interaction was examined by conducting a two-way mixed repeated measures ANOVA [injury × instant (pre and post)] for the variable average maximal sprint speed, FL, PA and MT in rest and active state. To determine the differences in femur length between the two groups, an independent t-test was used. An intra-individual comparison was performed between injured and contralateral limbs to compare the effects of the sprint task, using a two-way repeated measures ANOVA [injury × instant (pre and post)].

## 3. Results

No significant differences were observed for age (injured players: 25.79 ± 3.70; healthy controls: 25.79 ± 3.56 years; *p* = 1.000), height (injured players: 1.76 ± 0.06; healthy controls: 1.81 ± 0.09 m; *p* = 0.208) or weight (injured players: 73.53 ± 9.97; healthy controls: 76.21 ± 9.28 kg; *p* = 0.472). With respect to the femur length, no significant differences were seen between the previously injured players and healthy controls (injured players: 43.3 ± 1.75 cm; healthy controls: 43.19 ± 2.04 cm; *p* = 0.86; *d* = 0.06).

**Figure 2:**
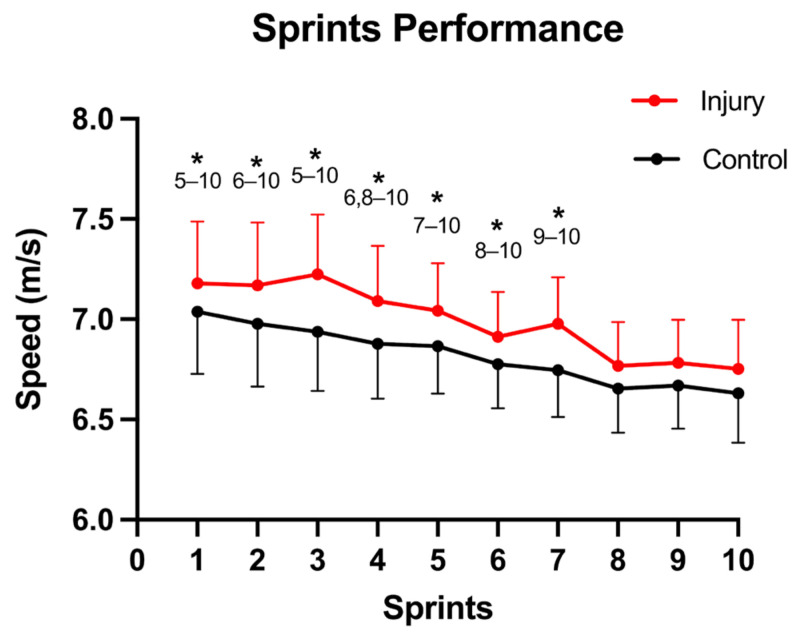
Acute effects of a sprint protocol on the average maximal speed injured players vs. healthy controls. Data are presented as mean ± standard deviation. * Significant difference between the current sprint and each subsequent sprint indicated.

Regarding the sprint performance, a significant decrease was seen for the average maximal sprint speed (*p* < 0.001; η^2^_p_ = 0.471) with a significantly higher speed for the injured players (injured: 7.00 ± 0.25 m/s; healthy controls: 6.82 ± 0.19 m/s; *p* = 0.026; η^2^_p_ = 0.155); however, no interaction was seen for instant × injury (*p* = 0.442; η^2^_p_ = 0.029) (Figure 2). For the comparison between injured players and healthy controls, the sprint task effects on BFlh FL, PA, MT and PT are shown in Table 1, reporting the ANOVA statistical analysis. In the resting state, a shorter FL (*p* = 0.014; η^2^_p_ = 0.196) and greater PA (*p* = 0.002; η^2^_p_ = 0.307) were seen for the previously injured players, with only the PA showing a significant increase between instants (*p* = 0.011; η^2^_p_ = 0.208), as well as an instant × injury interaction (*p* = 0.041; η^2^_p_ = 0.141). In the active state, a lower FL (*p* < 0.001; η^2^_p_ = 0.413) and PA (*p* < 0.001; η^2^_p_ = 0.368) were seen for the injured players. The peak torque produced in the MVIC demonstrated a significant decrease between instants (*p* < 0.001; η^2^_p_ = 0.399), but not for the injury factor, nor an instant × injury interaction.

For the comparison between injured vs. contralateral limbs, the sprinting effects on BFlh FL, PA and MT and peak torque are shown in Table 2, reporting the ANOVA statistical analysis. In the resting state, a lower FL was demonstrated for injured limbs (*p* = 0.012; η^2^_p_ = 0.519) with a decrease for both limb groups between instants (*p* < 0.001; η^2^_p_ = 0.799). The PA also presented a significant increase for both groups between instants (*p* < 0.001; η^2^_p_ = 0.732) with higher values for injured limbs (*p* = 0.006; η^2^_p_ = 0.582). In the active state, a lower FL was seen for injured limbs compared to contralateral limbs (*p* = 0.039; η^2^_p_ = 0.332) with no differences between instants nor instant × injury interaction. A higher PA was also shown for injured limbs (*p* = 0.018; η^2^_p_ = 0.412). The peak torque only showed a significant difference for the instant factor (*p* < 0.017; η^2^_p_ = 0.419). 

## 4. Discussion

In this study we examined BFlh architecture in passive (i.e., at rest) and active (i.e., at 20% of MVIC) conditions before and after a repeated sprint protocol in professional football players with and without previous HSI in the BFlh. To the best of our knowledge, this is the first retrospective study to examine the acute effects of a repeated sprint protocol on BFlh architecture in professional football players. The main findings were: (i) injury players have shorter FL and greater PA in passive and active conditions in relation to controls; (ii) injury limbs have shorter FL and greater PA in passive and active conditions in relation to their contralateral limb; (iii) the sprint task had limited impact on the BFlh architectural parameters.

Firstly, in relation to sprint performance, significant differences were seen between groups, with the injured football players displaying higher average maximal speed than the healthy control group. These results are in concordance with a previous study that reported higher maximal mean speed in using a protocol with the same distance, although with 6 repetitions and 90 s of rest between sets instead of the 10 repetitions and 30 s of rest between sets in this study [32]. On other hand, Røksund et al. (2017) reported that football players with a previous HSI showed a higher drop in speed during a repeated sprint test (8 × 20 m) with a slightly lower, but not significant, sprint time (5.25 s vs. 5.35 s) during one maximal sprint of 40-m. One possible reason for these contradictory results could be the heterogeneity between studies due to different protocols and samples, recovery, return to play and screening periods. Therefore, this warrants further investigation.

Regarding the PT, no significant differences were seen between injured and uninjured players, which agrees with a previous study that demonstrated (based on effect sizes and confidence intervals) similar short-length (hip = 0°, knee = 90°) isometric strength between previously injured limbs and uninjured contralateral limbs at assessment points beyond 7 days post-injury [33]. These results are similar in a meta-regression analysis of long-length (hip = 0°, knee = 0–15°) isometric strength, with no statistical differences between the two groups at assessment points beyond 7 days post-injury [34]. Despite the different protocol positions compared with our study (the hip at 0° and knee flexed at 30°), the results are consistent, indicating that time is fundamental for the recovery of isometric strength. Meanwhile, significant differences have been demonstrated between previously injured and uninjured contralateral limbs regarding their eccentric contraction strength during Nordic exercises [34]. This was further supported in a prospective study that showed no differences in isometric contraction strength but demonstrated differences in eccentric contraction strength between later-injured and uninjured players [15]. It should be noted that isometric contractions are extremely different from eccentric ones. Therefore, it is possible for the neuromuscular system to not be altered between these groups in isometric contractions and yet demonstrate differences in eccentric contractions. Interestingly, most injuries in the aforementioned prospective study occurred during the sprint (82%) in the BFlh (88%). Sprinting involves eccentric contractions in the hamstring group [15,35], and the BFlh is one of the greatest contributors in the late swing phase (where these injuries tend to occur more often [35]), it being relatively more eccentrically stretched in this phase [36]; this could be an explanation for the increased sprint-related BFlh injury among participants demonstrating statistically lower eccentric strength prior to injury. This warrants further investigation.

Furthermore, during eccentric actions, it was verified that the distance between the aponeuroses (averaged at various angular velocities) at a constant ankle joint angle was significantly greater compared with concentric muscle actions [37], this distance being possibly related to the reported smaller pennation angle and longer fascicles. It might be possible that with greater pennation angles and shorter fascicles, the distance between the aponeuroses would be even greater according to trigonometric equations. If so, the force produced by the fascicles would not be transmitted as efficiently given the orientation between the fascicles relative to the aponeuroses. However, these effects could be muscle dependent, and no differences were seen in the PT between all the groups, which can indicate that BFlh architecture is not a preponderant factor for isometric knee flexion torque production. Indeed, it should be noted that BFlh has a greater hip moment arm compared to the knee moment arm, being more sensitive to mobilization of hip flexion [38]; on the other hand, semitendinosus is being consistently identified as the greatest contributor to knee flexion [39,40,41]. Therefore, it would be interesting to analyze the behavior of the sprint protocol in hamstrings PT measured in hip extension. Future researchers should explore this approach.

Regarding the muscle architecture analysis, and in accordance with our initial hypothesis, shorter FL and greater PA were observed in the injured group when compared with both the healthy controls and contralateral limbs, thus indicating that these results are inter- and intra-individual dependent. The implications of shorter fascicles have also been studied, demonstrating that shorter fascicles can affect the joint angle where optimal force is produced during activity [42,43] and reduce the contraction velocity [44,45]. During repeated sprint running, hamstrings reach relatively long fascicle lengths. Thus, at relatively longer muscle lengths, shorter BFlh fascicles would present lower force-generating capacity compared with longer BFlh fascicles; however, it should be noted that in present study, previously injured players ran at higher speed than the uninjured, and higher FL was seen in the contralateral group. Since the study is retrospective, it can be suggested that the higher speed of the previously injured players (greater excursion and velocity capacity of the sarcomeres) and the higher FL in the contralateral limb favors the hypothesis that the reduced FL in previously injured players is a consequence of the injury. 

With respect to the comparison to the contralateral limb, these results are in line with the literature, with previous studies reporting shorter FL in the BFlh of players or athletes with previous injury [15,46,47]. Timmins et al. (2015) showed shorter BFlh fascicles in previously injured muscles compared with the contralateral uninjured muscles using a retrospective design, as in our study. However, the greater contralateral FL in the injured group indicates that it is not possible to determine whether this characteristic is a consequence of the injury or is a factor which predisposes to a greater risk of injury. Lima-E-Silva (2020) also reported that in professional senior and under-20 players, injured (in the previous season) players presented shorter BFlh FL compared with players without injury history. Additionally, injured players showed shorter BFlh FL in both limbs when compared with the threshold of 10.56 cm [15], although BFlh FL was symmetrical between limbs. Ribeiro-Alvares et al. (2020) also studied the prevalence of HSI risk factors in professional and under-20 male football players, finding that almost half of the sample of players had short BFlh relative FL (fascicle length/muscle length < 0.25) [15] and 30% of injury prevalence [47]. Overall, the greater contralateral FL in the present study possibly indicates that the shorter fascicles of football players can be a consequence of the injury and not a predictive measure.

In relation to the specificity of the task, in the present study, there was limited impact on the BFlh architectural parameters; specifically, a significant increase was seen only for PA in (i) the previously injured group in comparison with the healthy control group, and (ii) in the comparison between injured and contralateral limbs, via an exercise-induced decrease in FL for the contralateral with an increase in PA for both limbs in resting conditions. Repeated sprint exercise seems to decrease the net moments around the hip and knee, and the knee and hip peak joint angles [48]. Special consideration must be given to the fatigability associated with the repeated loading, as this has been suggested to increase the contractile compliance (decreasing muscle stiffness) as a result of repeated damage to the muscle with no changes in tendon compliance [49,50], suggesting that exercise induced changes in muscle-tendon function may lead to a greater strain in the BFlh. Moreover, muscle damage might be greater for individuals with shorter fascicle lengths, assuming the same (force) stress applied will induce a higher strain in BFlh. Therefore, these mechanical alterations due to lower FL are possible causes that place previously injured players at a higher injury risk of sustaining other HSI (with previous HSI being the most consistent risk factor), even though PT was similar in the uninjured individuals. However, no muscle and tendon stiffness was not measured and no differences were seen in FL after the repeated sprint task. Since the differences between instants are within the minimal detectable changes of the static technique (0.84 cm for FL and 1.5° for PA) [51] architectural changes following repeated sprints could be only statistically but not physiologically relevant. Moreover, it should be noted that while the task induced increases in PA and decreases in FL, the groups compared were not differentially affected by the task; the magnitude of the differences in FL and PA before the task were not statistically altered. This warrants further investigation.

Furthermore, we assessed the BFlh architecture using static technique; this technique allows for measurement during contractions. However, it is important to understand some methodological considerations when assessing muscle architecture, especially of the BFlh, which is heterogeneous, with the fascicles typically following a concave-to-convex path being differentially oriented along the muscle length. The FL estimation equation does not consider the different orientation of the fascicle, which might result in over- (for concave fascicles) or under-estimation (for convex fascicles) of the FL [28], implying that the assumptions with this type of methodology could affect the interpretation of the results. Additionally, it has been reported that transducer width is important in the linear extrapolation of the FL. Indeed, a 1 cm reduction in the transducer width would imply that approximately 11% of the FL would need to be estimated, which indicates an overestimation of the fascicle [28]. In our case, the probe used had 3.8 cm of width, which could overestimate ~24.2% of the FL when compared with Pimenta et al. (2018). It should also be noted that the visible portion of the fascicle in the linear extrapolation technique represents an extremely important factor in the equation that could increase or decrease the overestimation of the FL.

This study has some limitations. Firstly, these results can only be interpreted for senior professional male football players, since different results have been shown for different age groups [52], and the anatomical and biomechanical characteristics of running in females are different from those in males [53,54]. Secondly, the static technique has some limitations with respect to the extrapolation of the non-visible part of the fascicle, which is determined by the width of the transducer [28] and the equation used, which in this case overestimates FL by 0.5 cm, particularly with real FL above ~7 cm [31]; thus, these results should be analyzed carefully. Fascicle length will also depend on the femur length, where subjects with a larger femur would be expected to present larger fascicles. However, no significant differences were seen between groups, indicating that an anatomical criterion did not explain the differences between groups.

## 5. Conclusions

The present study provides evidence that injured football players in the BFlh are faster than the healthy control group, without difference in a mechanical parameter as PT. Regarding muscle architecture, in resting condition, injured players display shorter FL and greater PA than contralateral and healthy control limbs after a repeated sprint task. The same architectural differences were detected in the active condition after the repeated sprint task. Regarding the specificity of a repeated sprint exercise bout, the current study does not provide sufficient evidence to suggest the impact of the task on BFlh architectural parameters, nor that the task differentially impacts previously injured and uninjured groups. Overall, while one must take care when comparing architectural parameters between the limbs of different subjects; the FL differences observed between previously injured limbs and uninjured contralateral ones in both resting and active conditions could suggest the shorter FL to be a consequence of the injury rather than a predictive measure, as well as potentially indicating an increased risk of re-injury in the same limb. In fact, a history of HSI has been shown to be the most consistent risk factor for sustaining an HSI.

## Figures and Tables

**Table 1:** Acute effects of a sprint protocol on BFlh injured players vs. healthy controls on: fascicle length (FL), pennation angle (PA), muscle thickness (MT) and peak torque (PT) before and after the sprint task. Data are presented as mean ± standard deviation.

Injured Players vs. Healthy Controls											
		PRE SPRINT		POST SPRINT		Instant (Pre vs. Post)		Injury (Injured vs. Control)		Instant × Injury Interaction	
		Injured	Control	Injured	Control	*p*	η^2^_p_	*p*	η^2^_p_	*p*	η^2^_p_
**REST**	**FL** (cm)	7.40 ± 0.98	8.20 ± 0.80	7.02 ± 1.20	8.29 ± 1.38	0.298	0.039	0.014	0.196	0.103	0.092
	**PA** (°)	21.35 ± 2.67	18.47 ± 2.32	22.91 ± 3.09	18.65 ± 3.00	0.011	0.208	0.002	0.307	0.041	0.141
	**MT** (cm)	2.61 ± 0.28	2.58 ± 0.30	2.66 ± 0.26	2.60 ± 0.33	0.208	0.056	0.707	0.005	0.619	0.009
**ACTIVE**	**FL** (cm)	6.36 ± 1.21	7.89 ± 0.96	6.22 ± 1.13	7.86 ± 0.83	0.504	0.015	<0.001	0.413	0.677	0.006
	**PA** (°)	25.04 ± 3.34	20.28 ± 2.97	24.68 ± 4.06	20.60 ± 2.70	0.971	<0.001	<0.001	0.368	0.492	0.016
	**MT** (cm)	2.62 ± 0.27	2.69 ± 0.30	2.54 ± 0.24	2.73 ± 0.26	0.476	0.017	0.173	0.061	0.036	0.138
	**PT** (N⋅m)	141.17 ± 43.90	149.39 ± 35.45	132.54 ± 37.18	136.64 ± 32.27	<0.001	0.399	0.642	0.007	0.397	0.024

**Abbreviations:** FL, Fascicle length, PA, Pennation angle, MT, Muscle Thickness, PT, Peak Torque, p, *p* value, η^2^_p_, eta squared.

**Table 2:** Acute effects of a sprint protocol on BFlh injured vs. contralateral limbs on: fascicle length (FL), pennation angle (PA), muscle thickness (MT) and peak torque (PT) before and after sprint task. Data are presented as mean ± standard deviation.

Injured vs. Contralateral Limbs											
		PRE SPRINT		POST SPRINT		Instant (Pre vs. Post)		Injury (Injured vs. Healthy)		Instant × Injury Interaction	
		Injured	Contralateral	Injured	Contralateral	*p*	η^2^_p_	*p*	η^2^_p_	*p*	η^2^_p_
**REST**	**FL** (cm)	7.40 ± 0.98	9.14 ± 2.18	7.02 ± 1.20	8.36 ± 1.88	<0.001	0.799	0.012	0.519	0.394	0.082
	**PA** (°)	21.35 ± 2.67	18.81 ± 4.22	22.91 ± 3.09	20.14 ± 3.62	0.001	0.732	0.006	0.582	0.834	0.005
	**MT** (cm)	2.61 ± 0.28	2.77 ± 0.24	2.66 ± 0.26	2.75 ± 0.24	0.646	0.024	0.088	0.289	0.250	0.144
**ACTIVE**	**FL** (cm)	6.36 ± 1.21	7.73 ± 2.57	6.22 ± 1.13	7.60 ± 1.84	0.543	0.035	0.039	0.332	0.989	<0.001
	**PA** (°)	25.04 ± 3.34	22.06 ± 4.28	24.68 ± 4.06	21.23 ± 3.46	0.454	0.052	0.018	0.412	0.691	0.015
	**MT** (cm)	2.62 ± 0.27	2.70 ± 0.40	2.54 ± 0.24	2.60 ± 0.35	0.073	0.263	0.371	0.073	0.685	0.016
	**PT** (N⋅m)	141.17 ± 43.90	141.44 ± 36.59	132.54 ± 37.18	133.76 ± 35.53	0.017	0.419	0.857	0.003	0.735	0.011

**Abbreviations:** FL, Fascicle length, PA, Pennation angle, MT, Muscle Thickness, PT, Peak Torque, p, *p* value, η^2^_p_, eta squared.

## Data Availability

Not applicable.
